# In Vivo Activity of Metal Complexes Containing 1,10-Phenanthroline and 3,6,9-Trioxaundecanedioate Ligands against *Pseudomonas aeruginosa* Infection in *Galleria mellonella* Larvae

**DOI:** 10.3390/biomedicines10020222

**Published:** 2022-01-21

**Authors:** Megan O’Shaughnessy, Magdalena Piatek, Pauraic McCarron, Malachy McCann, Michael Devereux, Kevin Kavanagh, Orla Howe

**Affiliations:** 1 School of Biological and Health Sciences, Technological University Dublin-City Campus, D07 ADY7 Dublin, Ireland; megan.oshaughnessy@tudublin.ie; 2 Centre for Biomimetic and Therapeutic Research, FOCAS Research Institute, Technological University Dublin-City Campus, D08 CKP1 Dublin, Ireland; pauraic.mccarron@tudublin.ie (P.M.); michael.devereux@tudublin.ie (M.D.); 3 SSPC Pharma Research Centre, Department of Biology, Maynooth University, W23 F2H6 Kildare, Ireland; magdalena.piatek.2016@mumail.ie; 4 Chemistry Department, Maynooth University, W23 F2H6 Kildare, Ireland; malachy.mccann@nuim.ie

**Keywords:** *Pseudomonas aeruginosa*, *Galleria mellonella*, in vivo model, infection, metal complexes, 1,10-phenanthroline, antimicrobial resistance

## Abstract

Drug-resistant *Pseudomonas aeruginosa* is rapidly developing resulting in a serious global threat. Immunocompromised patients are specifically at risk, especially those with cystic fibrosis (CF). Novel metal complexes incorporating 1,10-phenanthroline (phen) ligands have previously demonstrated antibacterial and anti-biofilm effects against resistant *P. aeruginosa* from CF patients in vitro. Herein, we present the in vivo efficacy of {[Cu(3,6,9-tdda)(phen)_2_]·_3_H_2_O·EtOH}_n_ (Cu-tdda-phen), {[Mn(3,6,9-tdda)(phen)_2_]·_3_H_2_O·EtOH}_n_ (Mn-tdda-phen) and [Ag_2_(3,6,9-tdda)(phen)_4_]·EtOH (Ag-tdda-phen) (tddaH_2_ = 3,6,9-trioxaundecanedioic acid). Individual treatments of these metal-tdda-phen complexes and in combination with the established antibiotic gentamicin were evaluated in vivo in larvae of *Galleria mellonella* infected with clinical isolates and laboratory strains of *P. aeruginosa*. *G. mellonella* were able to tolerate all test complexes up to 10 µg/larva. In addition, the immune response was affected by stimulation of immune cells (hemocytes) and genes that encode for immune-related peptides, specifically *transferrin* and *inducible metallo-proteinase inhibitor*. The amalgamation of metal-tdda-phen complexes and gentamicin further intensified this response at lower concentrations, clearing a *P. aeruginosa* infection that were previously resistant to gentamicin alone. Therefore this work highlights the anti-pseudomonal capabilities of metal-tdda-phen complexes alone and combined with gentamicin in an in vivo model.

## 1. Introduction

Antimicrobial resistance (AMR) endangers the effective prevention and treatment of an expanding scope of infections caused by microorganisms that are no longer susceptible to the standard therapies used to manage them [1]. This presents a widespread and urgent threat to global health. Of particular concern are Gram-negative bacteria with surging resistance profiles especially to the last line of antibiotics [2]. The Gram-negative human pathogen, *Pseudomonas aeruginosa*, is a noteworthy contributor to elevated AMR prevalence and is frequently isolated from a diverse range of acute, chronic, and biofilm-associated infections. Most strains now present as multidrug-resistant which increase morbidity in infected patients, particularly those with cystic fibrosis (CF) [3,4,5]. The World Health Organization (WHO) deemed *P. aeruginosa* a critical priority pathogen urgently requiring the investigation of new agents to tackle its infections [6]. However, approximately 75% of the therapeutics under clinical assessment are derived from already established antimicrobials, of which multiple resistance mechanisms have been reported [7]. This highlights the clear necessity to investigate novel drugs with unique modes of action that can overcome these resistant pathways. Consequently, research into many non-antibiotic therapeutic approaches have been investigated including phage therapy [8], immunotherapy [9], and antimicrobial peptides [10,11,12]. In addition, inorganic complexes with 1,10-phenanthroline (phen) ligands have had a resurgence as possible alternatives or additives to established antimicrobial therapeutics [13,14,15]. Transition metal complexes containing phen or its derivatives have demonstrated promising therapeutic capabilities against bacteria [16,17], fungi [18,19,20], parasites [21,22,23], and viruses [24,25,26].

We recently demonstrated the activity profiles of manganese(II), copper(II), and silver(I) complexes incorporating 1,10-phenanthroline (phen) and 3,6,9-trioxaundecanedioate (tddaH_2_) against *P. aeruginosa* strains isolated from the lungs of CF patients [27]. These metal-tdda-phen complexes and the antibiotic gentamicin were assessed alone and combined on the planktonic growth, biofilm formation, and mature biofilm formation of the clinical isolates. The results showed the metal-tdda-phen complexes could prevent biofilm formation, with relation to mass and cellular viability, to a greater capacity than gentamicin across the clinical strains, and disturb mature biofilm. Gentamicin, in combination with the metal-tdda-phen complexes, displayed synergistic activity against the establishment of mature biofilms. This was supported by reducing the separate biofilm components examined, suggesting extracellular DNA (eDNA) and extracellular polysaccharides as potential molecular targets. The same complexes also previously demonstrated antitubercular [28] and antifungal [29] capabilities. The inclusion of the phen is paramount to their potency, with the addition of tddaH_2_ enhancing their water solubility and mode of action in various microbial cells. The ability to act on *P. aeruginosa* clinical isolates synergistically with gentamicin on mature biofilms prompted the in vivo studies, using larvae of the greater wax moth *Galleria mellonella*, reported within.

*G. mellonella* larvae are a valuable model system for assessing in vivo toxicity and mechanism of action studies of novel drugs. Low cost, ease of handling, and maintenance are desirable factors for using the larval model. They have a relatively short life cycle (40–60 days), and can survive at 37 °C which is an important attribute when assessing virulence and treatment of human pathogens [30]. Therefore, *G. mellonella* has been extensively utilized to evaluate the pathogenicity of bacterial and fungal pathogens [31,32,33], study biofilm formation [34], measure the in vivo toxicity of novel compounds [35,36], and determine the in vivo efficacy of established [37,38] and novel [19,39] antimicrobial agents. Moreover, studies assessing *G. mellonella* and mammalian models have reported a correlation in results obtained. Jander et al. [40] demonstrated similar virulence patterns in larvae and mice injected with *P. aeruginosa* mutants, and Brennan et al. [41] identified corresponding virulence patterns between *Candida albicans* in the aforementioned models. The generation of comparable data is due to the high degree of structural and functional analogies across human innate immunity and the insect immune response, composed of physical barriers and interconnected cellular and humoral responses [42,43]. Although the larvae do not have an adaptive immune system, they possess sophisticated systems for non-self-recognition and defence reactions. The hemolymph of *G. mellonella* consists of several hemocyte types, of which plasmatocytes and granular cells are the most common. These cells are similar to human neutrophils and macrophages, being involved in phagocytosis and encapsulation, resulting in an oxidation burst [44]. The humoral response consists of effector molecules such as antimicrobial peptides (AMPs), opsonins, and lytic enzymes that appear in the hemolymph to remove pathogens. In particular, AMPs have been found to destabilize membranes of invading microorganisms [45] as a mode of action. The humoral system also includes activating the phenoloxidase pathway, resulting in melanization species hydrogen peroxide and superoxide during the pathogen encapsulation [46].

Herein, we assess the pathogenicity of laboratory strains and clinical isolates of *P. aeruginosa* using this in vivo model of *G. mellonella* and the further therapeutic potential of manganese(II), copper(II), and silver(I) complexes incorporating 1,10-phenanthroline (phen) and 3,6,9-trioxaundecanedioate (tddaH_2_) ligands. Pathogenicity was characterized by the density of hemocytes and bacterial burden in the hemolymph of the larvae at several time points during infection with the clinical strains of *P. aeruginosa*. Simultaneously, the ability of the metal-tdda-phen complexes alone and combined with standard antibiotic gentamicin to interfere with the larval immune response was demonstrated. The antibacterial activity of the metal-tdda-phen complexes, alone and combined with gentamicin, against the *P. aeruginosa* laboratory strains and clinical isolates inoculated in *G. mellonella* are also presented.

## 2. Materials and Methods

### 2.1. Pseudomonas aeruginosa Strains and Culture Conditions

*Pseudomonas aeruginosa* (CF1, CF2, and CF3) isolated from cystic fibrosis (CF) patients at local Irish hospitals, with multidrug-resistant mechanisms [27], and standard laboratory strains ATCC 27853 and PAO1 were used in this study. Bacterial stocks were maintained on nutrient agar (Lab M) plates. Before experiments, a single colony was transferred to nutrient broth (Lab M) and grown at 37 °C and 200 rpm overnight. Bacterial cultures were adjusted by absorbance (OD_600_), harvested by centrifugation (2000× *g*), and washed twice with phosphate-buffered saline (PBS) prior to *G. mellonella* inoculation [47].

### 2.2. Galleria mellonella Larvae Monitoring

Larvae of the greater wax moth *G. mellonella* (Livefoods Direct Ltd., Sheffield, UK) were stored in wood chippings at 15 °C in darkness to prevent pupation. Larvae weighing 0.25 ± 0.05 g with no cuticle discoloration were selected. Ten healthy larvae per treatment and controls were used per experimental parameter. All experiments were performed independently on three separate occasions.

### 2.3. Test Complexes

The metal complexes used for this study, {[Cu(3,6,9-tdda)(phen)_2_]·_3_H_2_O·EtOH}_n_ **(Cu-tdda-phen)**, {[Mn(3,6,9-tdda)(phen)_2_]·_3_H_2_O·EtOH}_n_ **(Mn-tdda-phen)**, and [Ag_2_(3,6,9-tdda)(phen)_4_]·EtOH **(Ag-tdda-phen)** were prepared using previously reported methods [29,48]. In addition, the free ligands, 1,10-phenanthroline **(phen)** and 3,6,9-trioxaundecanedioic acid **(tddaH_2_)**, were also assessed. Gentamicin (Sigma-Aldrich, Kent, UK), an aminoglycoside antibiotic used to treat CF patients infected with *P. aeruginosa*, was also incorporated. All three clinical isolates demonstrated resistance to this antibiotic [27].

### 2.4. Galleria Mellonella Infection Studies with Pseudomonas aeruginosa Strains

#### 2.4.1. Bacterial Infection of *G. mellonella*

In the pathogenicity studies, ATCC 27853, PAO1, CF1, CF2, and CF3 were investigated by preparing a dilution series (3 × 10^0^ to 3 × 10^5^ CFU/mL) of each isolate, and ten larvae were inoculated through the last left pro-leg into the hemocoel using a Myjector U-100 insulin syringe (Terumo Europe NV, Leuven, Belgium) with 20 μL of washed cultures. Undisturbed larvae and larvae inoculated with PBS were utilized as controls. The injected larvae were placed in petri dishes containing wood shavings and were incubated at 37 °C. Mortality, cuticle discoloration, and response to touch were recorded 96 h post-injection. All experiments were performed independently on three separate occasions.

#### 2.4.2. Determination of Hemocyte Density

Hemocyte density was determined after the inoculation of larvae with laboratory strains ATCC 27853 (at 3 × 10^4^ CFU/mL) and PAO1 (at 3 × 10^0^ CFU/mL) and clinical isolates, CF1, CF2 (both at 3 × 10^3^ CFU/mL), and CF3 (at 3 × 10^0^ CFU/mL). A PBS control and undisturbed larvae were included. Changes in hemocyte density were measured at 0, 2, 4, 6, 12, and 24 h post-inoculation by piercing the back of the anterior end of five larvae (*n* = 3) with a sterile needle (25 gauge; BD Plastipak). Hemolymph was collected (50 µL) into a pre-chilled tube and diluted in cold PBS containing *N*-phenylthiourea (Sigma-Aldrich, Kent, UK) to reduce clotting and prevent melanization [49]. Cell density was calculated with a hemocytometer (Neubauer Tiefe) under the microscope.

#### 2.4.3. Determination of *G. mellonella* Hemolymph Burden of *P. aeruginosa*

Groups of five larvae (*n* = 3) were infected with 20 µL of ATCC 27853 (3 × 10^4^ CFU/mL), PAO1 (3 × 10^0^ CFU/mL) CF1 (3 × 10^3^ CFU/mL), CF2 (3 × 10^3^ CFU/mL), and CF3 (3 × 10^0^ CFU/mL) into the last left pro-leg and incubated for 0, 2, 4, 6, 12, and 24 h. After each time point, larval hemolymph (50 μL) was extracted and serially diluted in 450 μL of 0.9% NaCl in an iced tube. Each dilution was plated on *Pseudomonas* isolation agar (Lab M) and colonies were counted after 24 h at 37 °C. Data were expressed as viable CFU/mL.

### 2.5. Galleria mellonella Response to Metal-tdda-phen Complexes +/− Gentamicin

#### 2.5.1. Toxicity Studies

The Cu-tdda-phen, Mn-tdda-phen, and Ag-tdda-phen complexes were first tested in *G. mellonella* larvae to determine their levels of toxicity. Stock solutions of metal-tdda-phen complexes and gentamicin were diluted in sterile water and the starting materials, phen and tddaH_2_, were diluted in methanol (10%). Twenty µL were inoculated into the *G. mellonella* larvae through the last left pro-leg into the hemocoel using a Myjector U-100 insulin syringe (Terumo Europe NV, Leuven, Belgium). Undisturbed larvae, larvae inoculated with water, and larvae inoculated with methanol equivalent to the highest concentration present in the dilutions (10%) acted as experimental controls. The larvae were placed in Petri dishes containing wood shavings and incubated at 37 °C for 96 h. Mortality, cuticle discoloration, and response to touch were recorded every 12 h post-injection.

Once the toxicity profiles of each of the metal-tdda-phen complexes alone was established, they were individually assessed in combination with gentamicin. The working solutions 500, 200, and 100 µg/mL of both the metal-tdda-phen complexes and gentamicin were used in varying permutations by injection (20 µL) into the last left pro-leg. The larvae were placed in Petri dishes containing wood shavings and incubated at 37 °C and mortality, cuticle discoloration, and response to touch were recorded every 12 h post-injection.

#### 2.5.2. Determination of Hemocyte Density

Hemocyte densities were measured 2, 6, and 24 h post-inoculation with metal-tdda-phen complexes alone (200 and 750 µg/mL) and in combination with gentamicin (200 and 750 µg/mL). A PBS control and undisturbed larvae were included. Changes in hemocyte density were measured as previously described.

#### 2.5.3. Gene Expression of Immune-Related Genes

RNA extraction and quantification, followed by cDNA synthesis, was first carried out on *G. mellonella* larvae inoculated with the metal-tdda-phen complexes alone (750 µg/mL) or in combination with gentamicin (100 µg/mL) prior to real-time PCR for the expression of target immune-related genes. After 2, 6, and 24 h, five larvae (*n* = 3) were submerged in liquid nitrogen and ground to a fine powder. One mL of TRI Reagent (Sigma-Aldrich, Kent, UK) was added for the RNA extraction, and the mixture was transferred to a chilled tube before centrifugation at 2000× *g* for 2 min. The supernatant was brought to a fresh tube on ice and mixed with 200 µL of chloroform (ACS grade; Sigma-Aldrich, Kent, UK), incubated for 3 min at room temperature, before centrifuging again at 4 °C for 15 min at 12,000× *g*. The upper aqueous phase (200 µL) was carefully transferred into a tube, and RNA was precipitated with isopropanol (500 µL) (ACS grade; Sigma-Aldrich, Kent, UK). Tubes were then incubated at room temperature for 10 min and centrifuged at 4 °C for 10 min at 12,000× *g*. The RNA pellet was washed once in 1 mL of 75% (*v*/*v*) ethanol (Sigma-Aldrich, Kent, UK), allowed to dry and re-suspended in nuclease-free water. The RNA quantification was measured on a MultiskanTM GO (Thermo Scientific, Dublin, Ireland) UV spectrophotometer with the µDrop plate.

cDNA was then synthesized from the extracted RNA of each experimental condition using the cDNA synthesis kit (Quanta BioSciences, Gaithersburg, MD, USA). Each reaction was placed into the SimpliAmp Thermal Cycler (Applied Biosystems, Dublin, Ireland) for 5 min at 22 °C, 30 min at 42 °C, and 5 min at 85 °C, followed by samples being held at 4 °C before the quantitative real-time PCR (qRT-PCR). Primer sets (forward and reverse primer sequences detailed in Supplementary Table S1) [50] were obtained from Sigma-Aldrich and SYBR Green I (KAPA SYBR FAST; Sigma-Aldrich, Kent, UK) was used as the reaction probe. The samples were added to the 7500 Fast Real-Time PCR System (Applied Biosystems, Dublin, Ireland). Thermal cycling was initiated with a pre-incubation at 95 °C for 5 min, followed by amplification for 45 cycles of 95 °C for 10 s, 60 °C for 10 s, and 72 °C for 10 s, melting curve at 95 °C for 5 s, 65 °C for 1 min, and 95 °C for 10 s, and cooling at 40 °C for 10 s. The assay was performed in triplicate. The relative gene expression was calculated using the 2^−ΔΔCt^ method, and the Ct values of all immune-related genes analyzed were normalized against the expression of the reference gene (S7e).

### 2.6. Galleria mellonella Response to Pseudomonas aeruginosa Infection and Treatment with Metal-tdda-phen Complexes +/− Gentamicin

#### 2.6.1. Treatment of Metal-tdda-phen Complexes in *G. mellonella* Infected with *P. aeruginosa*

To ascertain the in vivo activity of metal-tdda-phen complexes alone, larvae were infected with each *P. aeruginosa* isolate (3 × 10^0^ CFU/mL for PAO1 and CF3, 3 × 10^3^ CFU/mL for CF1 and CF2, and 3 × 10^4^ CFU/mL for ATCC 27853) as described above and then administered with either Cu-tdda-phen, Mn-tdda-phen, Ag-tdda-phen or gentamicin (100–500 µg/mL) 1 h post-infection. Undisturbed larvae and larvae infected with the bacterial strains and PBS (untreated) were included as controls. All larvae were incubated at 37 °C and assessed every 12 h for a total of 96 h for mortality and melanization.

#### 2.6.2. Treatment of Metal-tdda-phen Complexes + Gentamicin in *G. mellonella* Infected with *P. aeruginosa*

Similarly to the above, larvae were infected with bacterial strains (3 × 10^0^ CFU/mL for PAO1 and CF3, 3 × 10^3^ CFU/mL for CF1 and CF2, and 3 × 10^4^ CFU/mL for ATCC 27853) and after 1 h post-inoculation, they were treated with a combination of metal-tdda-phen complex (100 µg/mL) and gentamicin (100 µg/mL). All larvae were incubated at 37 °C and assessed every 12 h for a total of 96 h.

### 2.7. Statistical Analysis

All experiments were performed in three independent experimental sets and the results are presented as the mean ± SE. All statistical analyses were performed with GraphPad Prism 9.0 (GraphPad Software Inc., San Diego, CA, USA). Survival curves of *G. mellonella* larvae experiments were generated using the Kaplan−Meier method and differences in survival between groups were calculated using the log-rank (Mantel-Cox) test, and Holm’s correction was applied for multiple comparisons. Two-way ANOVA was used to compare the hemocyte densities and gene expression studies.

## 3. Results

### 3.1. Response of Galleria mellonella to Pseudomonas aeruginosa Infection

**Figure 1:**
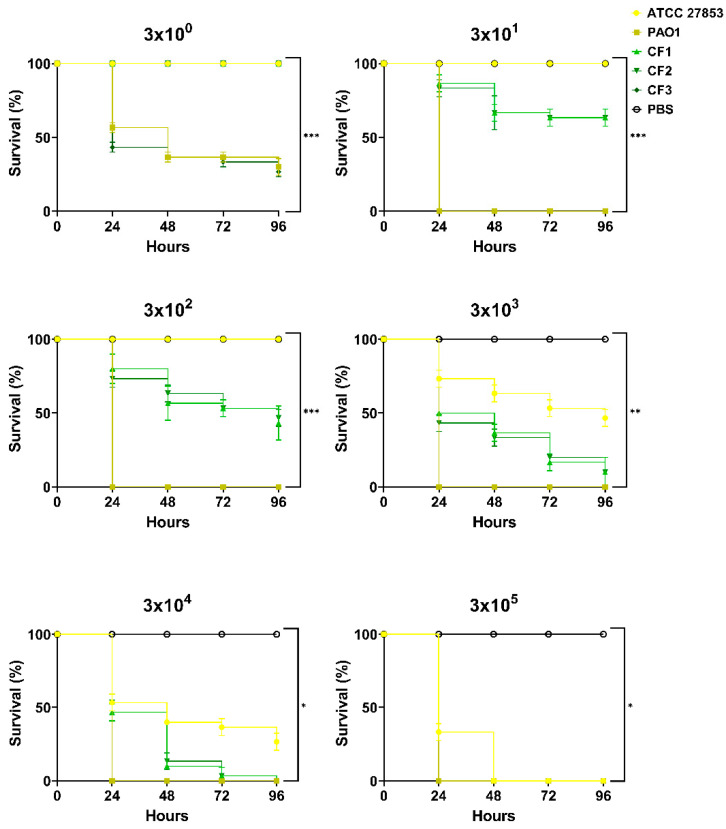
Kaplan–Meier survival distributions for each *P. aeruginosa* strain (ATCC 27853, PAO1, CF1, CF2, and CF3) assessed over varying inoculum doses (3 × 10^0^ to 3 × 10^5^ CFU/mL). Significance was assessed through the log-rank (Mantel–Cox) test, and Holm’s correction was applied for multiple comparisons (*: *p* < 0.05, **: *p* < 0.01, ***: *p* < 0.001).

The effect of infection with *P. aeruginosa* laboratory strains ATCC 27853 and PAO1, and clinical isolates CF1, CF2, and CF3, over a range of 3 × 10^0^ to 3 × 10^5^ CFU/mL, on survival of *G. mellonella* larvae is presented in Figure 1. Larval survival was affected in an inoculum-dependent manner during a 96 h incubation, with the greater CFU/mL injections causing greater larval death. All infected larvae died at the highest tested concentration (3 × 10^5^ CFU/mL) within a 48 h period. In the set of uninfected and PBS injected larvae used as a control, no death was observed. *G. mellonella* demonstrated the highest tolerance towards laboratory strain ATCC 27853, an inoculum size of 3 × 10^4^ CFU/mL induced mortality rates of 46.7 ± 3.3% after 24 h and 60 ± 3.3%, 63.7 ± 5.7%, and 73.3 ± 3.3% over the following days, and 3 × 10^3^ CFU/mL produced 26.7 ± 3.3% mortality after 24 h and 55 ± 5% by 96 h. Further dilutions (3 × 10^2^, 3 × 10^1^, 3 × 10^0^ CFU/mL) produced less than 10% mortality over the entire examined time course. Injection with clinical isolates CF1 and CF2 incited similar pathogenicity profiles. At 3 × 10^4^ CFU/mL, 53.3 ± 6.7% of larvae died after 24 h with the injection of CF1, while 46.7 ± 3.3% mortality was observed after infection with CF2. Over 90% death was recorded after 96 h of injection with either isolate. A ten-fold reduction in inoculum resulted in similar mortality after 24 h (50 ± 3.3% with CF1 and 56.7 ± 3.3% with CF2) with an 80 ± 5.7% mortality for both isolates by the end of the experiment. Injection with lower dilutions (3 × 10^0^; 3 × 10^1^; 3 × 10^2^ CFU/mL) resulted in a 0–26.7 ± 3.3% mortality after 24 h and 0–56.7 ± 6.7% after 96 h. Laboratory strain PAO1 and clinical isolate CF3 were the most virulent to the larvae, with a 20-µL inoculation of 3 × 10^1^ CFU/mL causing complete death after 24 h. This indicated the sensitivity of *G. mellonella* to *P. aeruginosa* infection, which has previously been reported [51,52,53]. There were significant differences (*p* < 0.05, *p* < 0.01, *p* < 0.001) between the virulence of the examined strains and an inoculum that caused over 50% mortality within 48 h but not 100% mortality within 24 h were chosen for subsequent studies.

### 3.2. Immune Response of Galleria mellonella to Pseudomonas aeruginosa Infection

**Figure 2:**
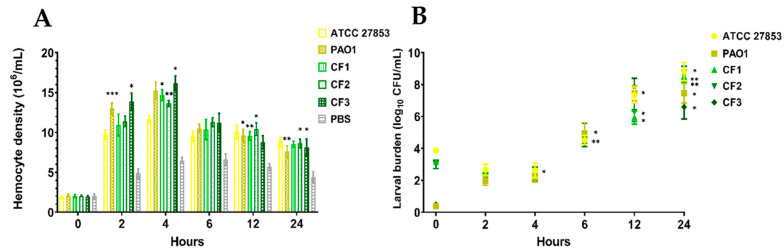
Following the inoculation of *G. mellonella* with *P. aeruginosa* strains ATCC 27583 (3 × 10^4^ CFU/mL), PAO1 (3 × 10^0^ CFU/mL), CF1 (3 × 10^3^ CFU/mL), CF2 (3 × 10^3^ CFU/mL), and CF3 (3 × 10^0^ CFU/mL), the (**A**) alteration in circulating hemocyte density and (**B**) bacterial burden, was assessed over a 24 h period. Data are presented as the mean ± SE of the three independent experiments. Statistical analysis was performed by comparing treatments to PBS injected controls at respective time points (**A**) and to prior time points (**B**) (*: *p* < 0.05, **: *p* < 0.01, ***: *p* < 0.001).

Alterations in the number of circulating immune cells (hemocytes) within *G. mellonella* have been used to indicate the larval immune response to a pathogen [54]. Drifting hemocytes are the initial responders to an infection, and their density can be enhanced by the stimulation of those attached to the internal wall of the haemocoel or bound to organs such as fat bodies [31]. Subsequent to the mortality studies, alterations in hemocyte density following inoculation with *P. aeruginosa* strains ATCC 27583 (3 × 10^4^ CFU/mL), PAO1 (3 × 10^0^ CFU/mL), CF1 (3 × 10^3^ CFU/mL), CF2 (3 × 10^3^ CFU/mL), and CF3 (3 × 10^0^ CFU/mL) were assessed over a 24-h period (Figure 2A). All inoculated larvae had increased levels of hemocytes relative to the initial injection (0 h). With strains PAO1 and CF3, the inoculum saw a significant (*p* < 0.001 and *p* < 0.05, respectively) increase in hemocytes (12.98 ± 0.73 and 13.86 ± 1.09 × 10^6^/mL, respectively) after 2 h. ATTC 27853 had a count of 9.69 ± 0.62 × 10^6^/mL at 2 h while CF1 had 10.93 ± 1.31 × 10^6^/mL and CF2 had 11.06 ± 1.00 × 10^6^/mL. Infections with all strains saw a spike in hemocyte populations after 4 h. The greatest response was observed in larvae injected with CF3 (16.17 ± 0.94 × 10^6^/mL, *p* < 0.001) followed by PAO1 (15.27 ± 1.04 × 10^6^/mL), CF1 (14.67 ± 0.66 × 10^6^/mL, *p* < 0.05), CF2 (13.62 ± 0.38 × 10^6^/mL, *p* < 0.01), and ATCC 27853 (11.64 ± 0.51 × 10^6^/mL). After 24 h of monitoring, hemocytes populations had decreased but remained elevated compared to their initial levels (0 h). Larvae infected with PAO1 (7.59 ± 0.71 × 10^6^/mL, *p* < 0.01), CF2 (8.61 ± 0.55 × 10^6^/mL, *p* < 0.05), and CF3 for 24 h (8.12 ± 1.06 × 10^6^/mL, *p* < 0.05) showed a significant increase in hemocyte density compared to the controls, ATCC 27853 (8.81 ± 0.57 × 10^6^/mL, *p* > 0.05) and CF1 (8.51 ± 0.39 × 10^6^/mL, *p* > 0.05) were not significant.

The infection process was also observed by enumerating *P. aeruginosa* CFU in the hemolymph of the infected larvae. Larvae were infected with ATCC 27853 (3 × 10^4^ CFU/mL), PAO1 (3 × 10^0^ CFU/mL), CF1 (3 × 10^3^ CFU/mL), CF2 (3 × 10^3^ CFU/mL), and CF3 (3 × 10^0^ CFU/mL) and monitored over 24 h (Figure 2B). With initial time points (0 to 4 h), a decrease in viable bacterial cells was observed. Bacterial burden in larvae infected with all bacterial strains increased from 4 and 24 h analysis points, strains ATCC 27853, PAO1, CF1, and CF2 increased to a median value >5 log_10_ CFU/mL while CF3 increased to a median value >4 log_10_ CFU/mL.

### 3.3. Galleria mellonella Response to Metal-tdda-phen Complexes

**Figure 3:**
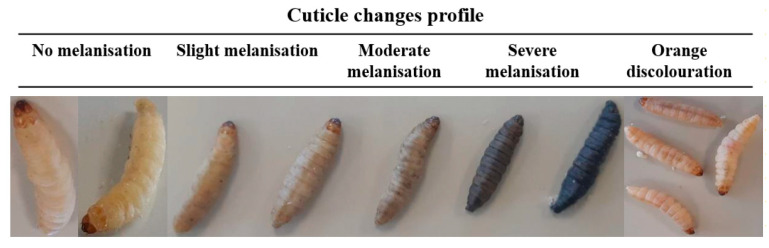
*G. mellonella* representing the different levels of melanization and cuticle discoloration.

*G. mellonella* larvae were exposed to Mn-tdda-phen, Cu-tdda-phen, Ag-tdda-phen, and gentamicin, and mean mortality (%) was assessed over 72 h (Table 1). No mortality was recorded after incubation of larvae with solvent (10% methanol), PBS inoculated larvae, and larvae that were undisturbed (data not shown) at any time point. However, larvae exposed to the solvent control did demonstrate slight to moderate melanization as presented in Figure 3. All larvae tolerated 2–10 µg/larvae (1.6–13.59 µM) of the metal-tdda-phen complex with no mortality observed over the entire experiment. Similarly, complexes Mn-tdda-phen and Ag-tdda-phen induced no mortality at 15 µg/larvae (20.39 and 12.5 µM, respectively) while doubling the concentration (40.78 and 24.9 µM, respectively) resulted in 6.66 ± 5.77% death for the former and 23.33 ± 5.77% death for the latter, after exposure for 72 h. Cu-tdda-phen was the most toxic to the larvae, a concentration of 15 µg/larvae (20.15 µM) resulted in a mortality rate of 53.33 ± 5.77% after 72 h and increasing the dose to 30 µg/larvae (40.3 µM) saw complete death of all tested larvae after the same amount of time. It is well known that copper is highly toxic to mammals, and similar studies investigating Cu-phen complexes and their derivatives have also highlighted their lethality towards *G. mellonella* [28,35]. Although lower concentrations of Cu-tdda-phen did not induce mortality, moderate to severe melanization was observed. Gandra et al. [39] investigated the toxicity of one copper(II)-phen, seven manganese(II)-phen, and three silver(I)-phen-complexes towards *G. mellonella*, and of this panel Mn-tdda-phen (chelate 8), Cu-tdda-phen (chelate 1), and Ag-tdda-phen (chelate 10) were included. The group also reported the low mortality rate induced in larvae by Mn-tdda-phen (13.33 ± 5.77%) and Ag-tdda-phen (33.33 ± 5.77%) at high concentrations (30 µg/larvae) and the toxicity of Cu-tdda-phen (100% mortality), corroborating the results obtained in this study.

**Figure 4:**
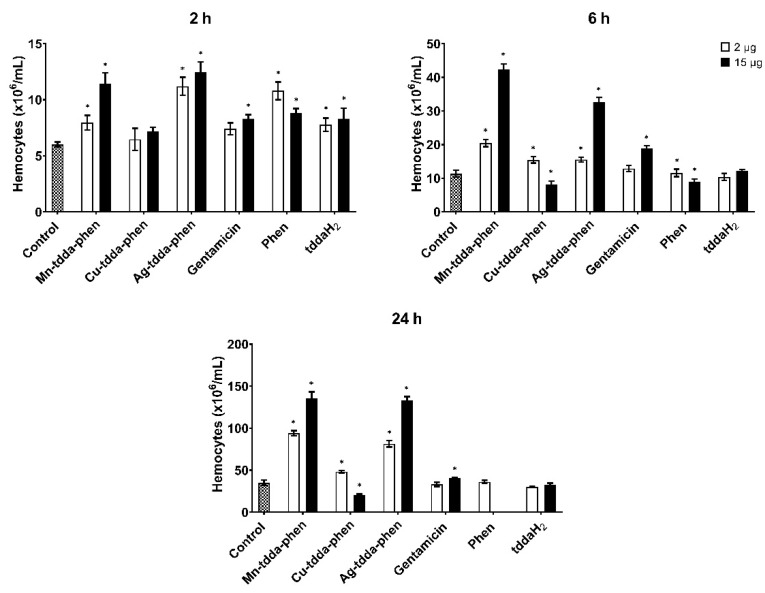
Immunomodulation induced by the metal-tdda-phen complexes and gentamicin (2 and 15 µg/larvae) in *G. mellonella* after 2, 6, and 24 h post-injection. * indicate significant differences in relation to the PBS injected control (*p* < 0.05).

Injection of the control antibiotic, gentamicin at 30 µg/larvae (52.1 µM) induced 13.33 ± 5.77% mortality after 48 h. Surviving larvae presented with slight to moderate melanization and some were slow to respond to stimulation. In addition, the larvae were injected with the metal-tdda-phen complexes starting materials, 3,6,9-trioxaundecanedioic acid (tddaH_2_) and 1,10-phenanthroline (phen) to ensure the recorded effects were not a result of the free ligands alone but the complex itself. The highest dose (30 µg/larvae, 135 µM) of tddaH_2_ saw 46.7 ± 5.8% of the inoculated larvae perish while 10 µg/larvae (45 µM) resulted in a mortality of 53.3 ± 5.8% after the initial 24 h. Kellett et al. [55] also demonstrated that *G. mellonella* exposed to high concentrations of phen (5000 and 2000 μg mL^−1^) had poor tolerance (100% and 90% mortality, respectively). Interestingly, the *G. mellonella* larvae that were exposed to lower doses of phen (2–4 µg/larvae, 11.1–2.2 µM), although they survived, showed an orange discoloration of the cuticle post-injection (Figure 3). It was postulated that the orange discoloration was a result of the phen interfering with the copper containing enzyme phenoloxidase that drives melanin synthesis [43]. To identify whether metal-tdda-phen complexes induced an immunomodulatory effect, larval hemocytes were withdrawn and counted after exposure to a low (2 µg/larvae) and high (15 µg/larvae) dose that did not induce 100% mortality. At 2 h, both low and high doses prompted similar responses in subjected larvae: Ag-tdda-phen (11.20 ± 0.47 × 10^6^/mL and 12.44 ± 0.54 × 10^6^/mL, respectively), Mn-tdda-phen (7.95 ± 0.37 × 10^6^/mL and 11.43 ± 0.56 × 10^6^/mL, respectively), phen (10.8 ± 0.46 × 10^6^/mL and 8.8 ± 0.24 × 10^6^/mL, respectively), and tddaH_2_ (7.78 ± 0.34 × 10^6^/mL and 8.30 ± 0.55 × 10^6^/mL, respectively) showed a significant (*p* < 0.05) increase compared to the control (Figure 4). After an extended exposure to 15 µg/larvae to 6 h, Mn-tdda-phen (42.34 ± 0.95 × 10^6^/mL) and Ag-tdda-phen (32.71 ± 0.75 × 10^6^/mL) significantly enhanced hemocyte density that continued to the 24 h time point (135.82 ± 4.29 and 133.14 ± 2.59 × 10^6^/mL, respectively). This demonstrates that at higher concentrations, these metal-tdda-phen complexes induce a priming effect within the insects. There were no hemocytes when phen was assessed at 15 µg/larvae after 24 h, and reduced hemocytes when Cu-tdda-phen (20.66 ± 0.61 × 10^6^/mL) was examined at the same concentration. The toxicity of these compounds to larvae at this concentration could be responsible for this response.

**Figure 5:**
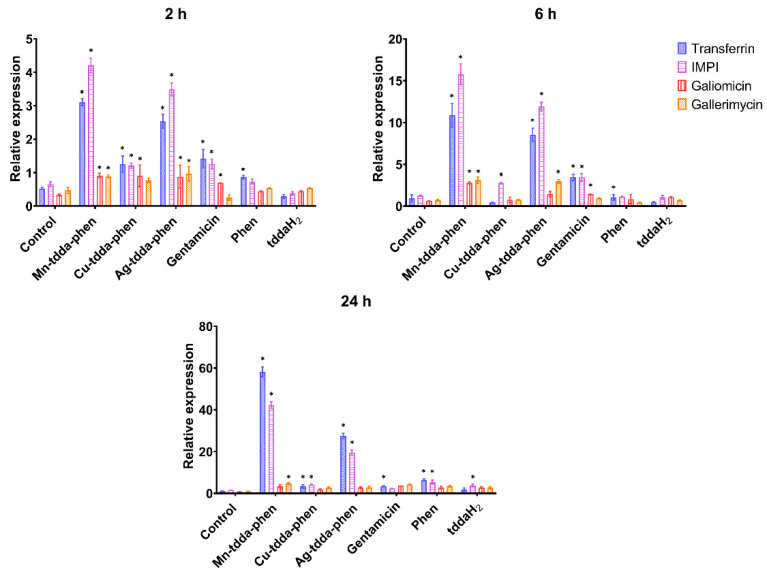
Relative expression of genes involved in the immune response of *G. mellonella* when exposed to metal-tdda-phen complexes, gentamicin and phen after 2, 6, and 24 h post-injection. * indicate significant differences to the PBS injected control (*p* < 0.05).

Larvae treated with Mn-tdda-phen, Cu-tdda-phen, Ag-tdda-phen, and gentamicin (15 µg/larvae) were incubated for 2, 6, and 24 h prior to assessment of immune related gene expression. *Transferrin* (iron-binding protein)*, IMPI* (inducible metallo-proteinase inhibitor)*, galiomicin* (defensin), and *gallerimycin* (cysteine-rich antifungal peptide) genes were normalized against the expression of *S7e* (reference gene) and larval treatments were compared to the PBS injected control and are presented in Figure 5. Expression of *transferrin* and *IMPI* encoding genes were significantly (*p* < 0.05) upregulated by Mn-tdda-phen and Ag-tdda-phen across all time points. A time-dependent induction of both genes was observed reaching the maximum 24 h after injection. This suggests that these metal-tdda-phen complexes are initiating an immune response in the larvae.

### 3.4. Galleria mellonella Response to Metal-tdda-phen Complexes and Gentamicin

After toxicity studies of the metal-tdda-phen complexes and gentamicin as single agents, the dual administration of the complexes and antibiotic were assessed within the larval model. The lowest examined concentrations that did not induce mortality (Table 1) were examined in varying permutations of 2–10 µg/larvae and observed over three time points (24, 48, and 72 h) (Table 2). Overall, the combination of agents, even at lower doses, enhanced toxicity towards the larvae when compared to their toxicity as singular drugs. The highest dose of gentamicin (10 µg/17.4 µM) with Mn-tdda-phen (10 µg/13.59 µM), Cu-tdda-phen (10 µg/13.41 µM), and Ag-tdda-phen (10 µg/8.3 µM) resulted in 86.7, 100, and 93.3% mortality, respectively, after 72 h. While the highest dose of gentamicin (10 µg/17.4 µM) with the lowest dose of Mn-tdda-phen (2 µg/2.71 µM) and Ag-tdda-phen (2 µg/1.6 µM) produced mortality rates of 46.7 and 53.3%, respectively, the combination with Cu-tdda-phen (2 µg/2.68 µM) incited 83% mortality. The lowest concentration of gentamicin (2 µg/2.35 µM) with both Mn-tdda-phen (2 µg/2.71 µM) and Ag-tdda-phen (2 µg/1.6 µM) induced complete survival of all injected larvae and with Cu-tdda-phen (2 µg/3.5 µM), 26.7% mortality was noted. A high and low and a low and high dose of gentamicin with both Mn-tdda-phen and Ag-tdda-phen exerted a similar toxicity profile in larvae (43.3–53.3% mortality) suggesting that a combination of agents overstimulate the animal, irrespective of the higher concentration. In contrast, a higher dose of Cu-tdda-phen (10 µg/13.41 µM) with a lower dose of gentamicin (2 µg/2.35 µM) induced an elevated mortality (83.3%) than the reverse (73.3%), suggesting that Cu-tdda-phen is driving the toxicity towards *G. mellonella*.

**Figure 6:**
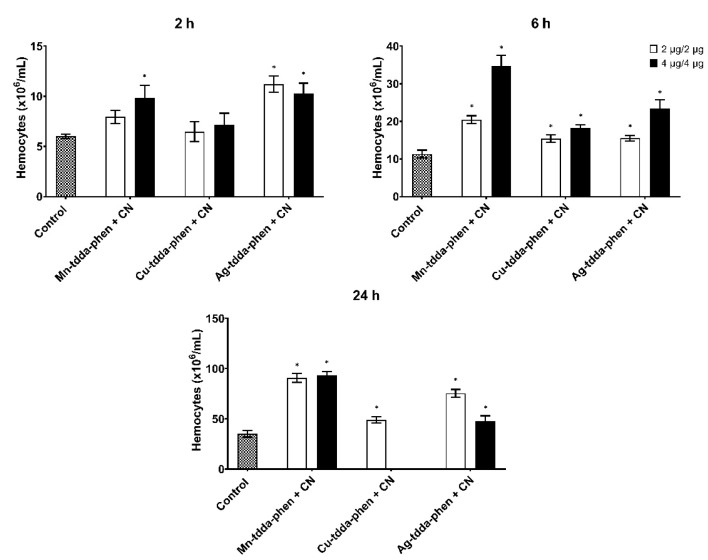
Immunomodulation induced by the metal-tdda-phen complexes in combination with gentamicin (CN) (2 µg and 2 µg/larvae, and 4 µg and 4 µg/larvae) in *G. mellonella* after 2, 6, and 24 h post-injection. * indicate significant differences in relation to the PBS injected control (*p* < 0.05).

To further investigate the combined effect of metal-tdda-phen complexes and gentamicin on the immune system of the larvae, hemocytes were extracted and enumerated (Figure 6). The combination of Mn-tdda-phen (2 (2.71 µM)—4 µg (5.42 µM)) with gentamicin (2 (3.5 µM)—4 µg (6.9 µM)) elicited the greatest hemocyte response at 2 (7.59 ± 0.38 and 9.82 ± 0.74 × 10^6^/mL, respectively), 6 (20.44 ± 0.61 and 34.67 ± 1.64 × 10^6^/mL, respectively), and 24 h (90.61 ± 2.56 and 93.48 ± 2.04 × 10^6^/mL, respectively). Administration of Cu-tdda-phen with gentamicin to larvae produced similar hemocyte densities at both low (2 (2.71 µM) + 2µg (3.5 µM)) and high doses (4 (5.36 µM) + 4 µg (6.9 µM)) at 2 (6.48 ± 0.57 and 8.14 ± 1.24 × 10^6^/mL, respectively) and 6 h (15.44 ± 0.56 and 18.21 ± 0.52 × 10^6^/mL, respectively). After 24 h, a hemocyte count of Cu-tdda-phen and gentamicin could not be determined due to the high mortality rate. Unlike the other combinations, Ag-tdda-phen and gentamicin induced a more pronounced hemocyte response at lower concentrations than higher after 2 (11.20 ± 0.47 and 10.29 ± 0.59 × 10^6^/mL, respectively) and 24 h (75.28 ± 2.28 and 47.92 ± 2.88 × 10^6^/mL, respectively).

**Figure 7:**
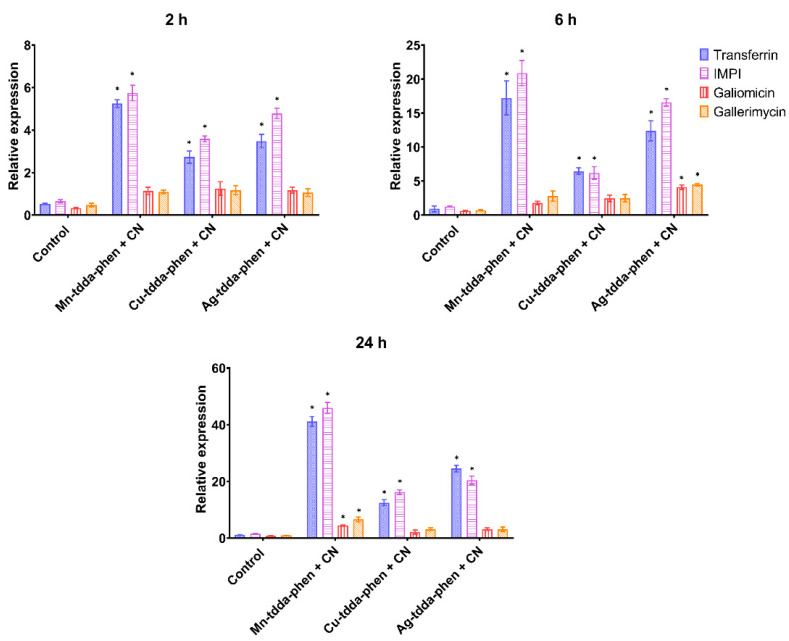
Relative expression of genes involved in the immune response of *G. mellonella* when exposed to metal-tdda-phen complexes in combination with gentamicin (CN) after 2, 6, and 24 h post-injection. * indicate significant differences to the PBS control (*p* < 0.05).

The expression of *transferrin* (iron-binding protein)*, IMPI* (inducible metallo-proteinase inhibitor)*, galiomicin* (defensin) and *gallerimycin* (cysteine-rich antifungal peptide) genes was assessed after larvae were exposed to a combination of metal-tdda-phen complex (2 µg/larvae) and gentamicin (2 µg/larvae) for 2, 6, and 24 h (Figure 7). Similar responses were observed to the metal-tdda-phen complexes and gentamicin as single agents (Figure 5), with significant (*p* < 0.05) upregulation of *transferrin* and *IMPI* encoding genes across all time points.

### 3.5. Effect of Metal-tdda-phen Complexes in Treating Pseudomonas aeruginosa Infection in Galleria mellonella +/− Gentamicin

After screening the bacterial strains in the larval model, an infective dose was determined for each strain. Larvae were inoculated with ATCC 27583 (3 × 10^4^ CFU/mL), PAO1 (3 × 10^0^ CFU/mL), CF1 (3 × 10^3^ CFU/mL), CF2 (3 × 10^3^ CFU/mL), and CF3 (3 × 10^0^ CFU/mL), and subsequently received a single dose of the metal-tdda-phen complexes alone (2–10 µg/larvae) (1.6–13.59 µM), gentamicin (2–10 µg/larvae) (3.5–17.4 µM) alone, or the metal-tdda-phen complex (1 µg/larvae) (831.3 nM–1.36 µM) in combination with gentamicin (1 µg/larvae) (1.74 µM) 1 h post-infection.

**Figure 8:**
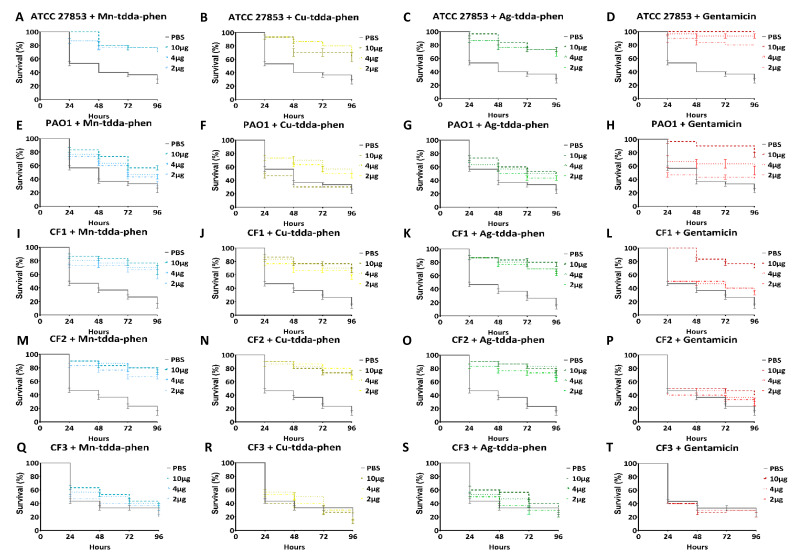
Survival (%) of *G. mellonella* inoculated with *P. aeruginosa* strains, ATCC 27853 (**A**–**D**), PAO1 (**E**–**H**), CF1 (**I**–**L**), CF2 (**M**–**P**), CF3 (**Q**–**T**), and treated with 2–10 µg/larvae of Mn-tdda-phen (**A**,**E**,**I**,**M**,**Q**), Cu-tdda-phen (**B**,**F**,**J**,**N**,**R**), Ag-tdda-phen (**C**,**G**,**K**,**O**,**S**), and gentamicin (**D**,**H**,**L**,**T**) over 96 h.

The effect of single doses of metal-tdda-phen complexes or gentamicin on survival of *G. mellonella* inoculated with *P. aeruginosa* strains, ATCC 27853 (A-D), PAO1 (E-H), CF1 (I-L), CF2 (M-P), CF3 (R-U), are presented in Figure 8. Overall, the exposure to a metal-tdda-phen complex increased survival in infected larvae. Gentamicin (10 µg/larvae) (17.4 µM) was the most effective at treating larvae infected with ATCC 27853 which had no mortalities after 96 h when compared to the PBS control (73.33 ± 3.3% mortality). All metal-tdda-phen complexes decreased mortality (26.7–36.7%) at the same concentration and time point. When larvae were inoculated with PAO1, a more virulent strain, mortality increased, except for Cu-tdda-phen treatment. *G. mellonella* presented with a metal-tdda-phen complex or gentamicin increased survival in a dose-dependent manner. Larvae administered 10 µg of gentamicin (17.4 µM) had a 23.3 ± 3.3% mortality rate. Those exposed to Mn-tdda-phen (13.59 µM) and Ag-tdda-phen (8.3 µM) at 10 µg/larvae prolonged survival of PAO1 infected larvae. At 48 h, the mortality rate was 26.7 ± 3.3% of larvae presented with Mn-tdda-phen and 40 ± 5.8% of larvae subjected to Ag-tdda-phen, compared to 66.7 ± 6.7% of larvae that received PBS. *G. mellonella* that encountered 10 µg (13.41 µM) of Cu-tdda-phen showed a higher mortality rate (70 ± 0%) at 48 h than those that were given PBS. Due to the toxicity of this complex in larvae, the stress of both seemed to elevate mortality. However, a lower dose of 4 µg/larvae produced a mortality rate of 36.7 ± 3.7 at 48 h. This was expected as Mn-tdda-phen and Ag-tdda-phen presented with lower toxicity towards *G. mellonella* (Table 2) and induced the immune response (Figure 6) of the larvae, compared to their copper equivalent. We have previously reported the susceptibility profiles of laboratory strains and clinical isolates CF1-CF3 to gentamicin [27] in vitro. ATCC 27853 and PAO1 were susceptible to gentamicin with MICs of 1 (1.7 µM) and 2 µg/mL (3.5 µM), respectively, while all the clinical isolates (MICs of 8 (13.9 µM) to over 256 µg/mL (445 µM)) were deemed resistant to the antibiotic. Similarly in the *G. mellonella* model, the antibiotic efficacy against the clinical isolates decreased compared to the laboratory strains. Larvae infected with CF1, CF2, and CF3 and subsequently treated with the highest dose of gentamicin (10 µg/larvae) (17.4 µM) saw survival recorded at 30 ± 0%, 70 ± 5.8%, and 76.7 ± 3.3%, respectively, after 96 h. At the lowest investigated concentrations of metal-tdda-phen complexes (2 µg/larvae) (1.6–2.71 µM), mortality rates were noted at 33.3–43.3% for CF1 and CF2. This activity was not maintained with CF3 however, treatment of metal-tdda-phen complexes extended survival when compared to the PBS treated larvae. After 48 h, larvae treated with Mn-tdda-phen (5.42 µM), Cu-tdda-phen (5.36 µM), and Ag-tdda-phen (3.3 µM) at 4 µg/larvae had mortality rates of 50 ± 0%, 60 ± 5.8%, and 53.3 ± 3.3%, respectively, compared to a 66.7 ± 3.3% of larvae that received PBS. Again, this activity profile draws similarities to the result obtained when assessed in vitro; metal-tdda-phen complexes were the most active against CF1 and CF2 (with MICs of 8–16 µg/mL); however, a higher concentration was needed to inhibit CF3 (MICs of 64–128 µg/mL).

**Figure 9:**
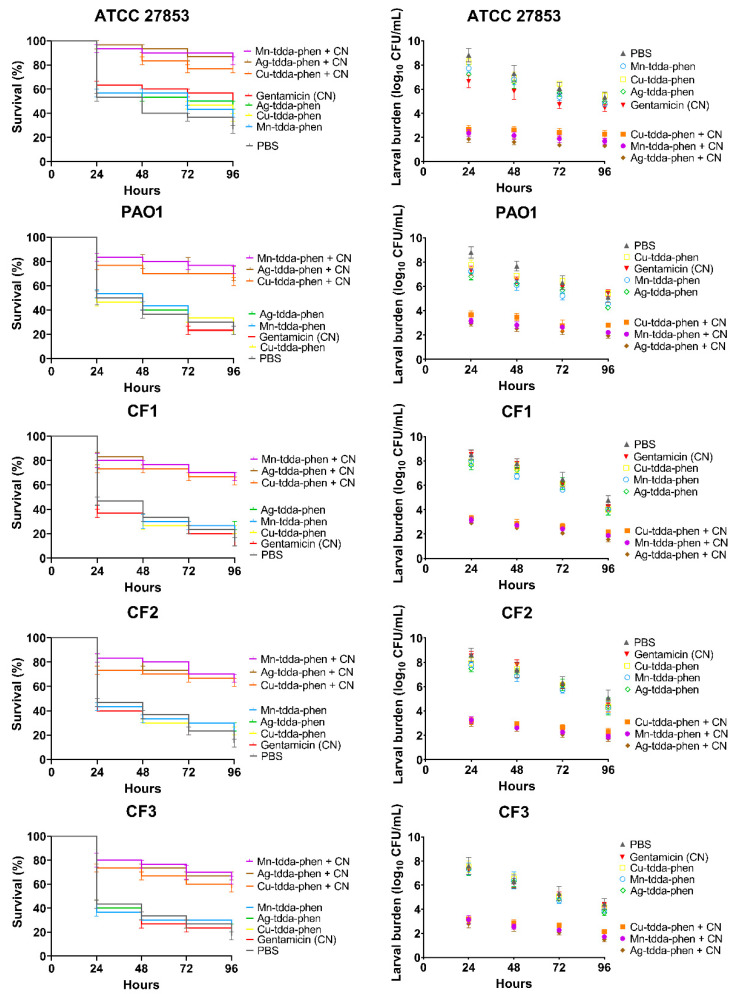
Effect of treatment with Mn-tdda-phen, Cu-tdda-phen, and Ag-tdda-phen alone (1 µg/larvae) and in combination with gentamicin (CN) (1 µg/larvae) infected with ATCC 27853, PAO1, CF1, CF2, and CF3 on survival (**left**) and larval bacterial burden (**right**).

In an effort to improve therapeutic outcome, many clinicians recommend dual combinations of antibiotics to increase the likelihood of achieving appropriate therapy of multidrug-resistant *P. aeruginosa* infections [56]. We also demonstrated synergistic activity against *P. aeruginosa* between all three complexes and gentamicin in vitro [27]. The efficacy of metal-tdda-phen complexes in combination with gentamicin was measured in *G. mellonella* larvae infected with ATCC 27583 (3 × 10^4^ CFU/mL), PAO1 (3 × 10^0^ CFU/mL), CF1 (3 × 10^3^ CFU/mL), CF2 (3 × 10^3^ CFU/mL), and CF3 (3 × 10^0^ CFU/mL). A single dose of both Mn-tdda-phen (1.36 µM) and gentamicin (1.74 µM), Cu-tdda-phen (1.34 µM) and gentamicin, and Ag-tdda-phen (868.5 nM) and gentamicin at 1 µg/larvae was administered 1 h post-infection and monitored for 96 h (Figure 9). This concentration was chosen as no mortality was observed when dual administered to larvae (data not shown). Overall, a combination of both drugs outperformed either as a single entity. Of the combinations, larvae that received Mn-tdda-phen and gentamicin and Ag-tdda-phen and gentamicin, had the lowest mortality across all strains. Both combinations decreased mortality by 50–53.3%, compared to the PBS treated larvae, while Cu-tdda-phen and gentamicin decreased mortality by 43.3–50%.

To complement the survival data, the effect of these combinations was also assessed through analyzing larval bacterial burden of *P. aeruginosa* compared to the effect of their constituent mono-therapies and PBS treated larvae (Figure 9). Analysis after 24 h demonstrated that all combinations significantly reduced the bacterial population in the infected larvae. *G. mellonella* infected with ATCC 27853 or PAO1 and subsequently treated with metal-tdda-phen complexes in combination with gentamicin had a 6–7 log_10_ CFU/mL reduction in comparison to PBS treated larvae. Activity was maintained across clinical isolates, CF1–CF3, which had 4–6 log_10_ CFU/mL in circulating cells that were exposed to both metal-tdda-phen complexes and gentamicin.

## 4. Discussion

*Pseudomonas aeruginosa* is a versatile opportunistic pathogen that causes severe clinical complications due to its large genome that harbours an extensive arsenal of virulence factors and antibiotic resistance determinants [57]. The bacterium is well reported to swiftly adapt to conditions in the airway with exceptional metabolic flexibility and ability to evade host immune attack [58]. The presence of *P. aeruginosa* is a particular threat for cystic fibrosis (CF) patients. The deleterious impact that chronic infection has on lung function in CF has been well described and often indicates poor clinical outcomes. *P. aeruginosa* infections are becoming more challenging to treat due to the inherent resistance to many antibiotics, and the prevalence of multidrug-resistance is increasing worldwide [59]. Moreover, persistent *P. aeruginosa* infections in CF patients is due to the bacteria’s affinity to biofilm formation which are exceedingly more resistant to treatment than their planktonic form [60,61]. Thus, our previous report on the metal-tdda-phen complex capabilities against established mature biofilms and synergistic activity with gentamicin warranted the in vivo studies to further explore the hypothesis that these complexes could be potential therapeutics for treating *P. aeruginosa* infection as a mono- or combination-therapy.

The use of *Galleria mellonella* larvae as a model to study pathogenicity and virulence, the toxicity of novel complexes, and their efficacy as therapies is now well established. Entomopathogenic strains, such as ATCC 27853 and PAO1, and clinical isolates of *P. aeruginosa*, are highly virulent in *G. mellonella* with lethal doses ranging from 2 to 100 CFU, killing infected larvae within 24–48 h [40,51,62,63,64]. Similar pathogenicity profiles were observed in this study. For instance, PAO1 and CF3 resulted in complete larvae death after 24 h at 30 CFU. Mortality assays demonstrated that the metal-tdda-phen complexes are well tolerated by *G. mellonella* up to 10 µg per exposed larvae. At the highest examined concentration (30 µg/larvae), Cu-tdda-phen was extremely toxic to the larvae while Mn-tdda-phen was the least toxic. This was also previously reported within our research group [39] demonstrating the reproducibility of this model. *G. mellonella* have been employed to evaluate the toxicity of a range of agents and the results have shown a strong correlation to those generated utilizing mammalian models. Maguire et al. reported comparable toxicology data (LD_50_) of food preservatives between insect larvae and rats; consequently, Mn-tdda-phen and Ag-tdda-phen complexes could be well tolerated by a mammalian model [65]. Furthermore, Mn-tdda-phen demonstrated immunomodulation properties by simulating hemocyte density and immune-related genes, specifically antimicrobial peptides (AMPs) *transferrin* (iron-binding protein) and *IMPI* (inducible metallo-proteinase inhibitor). AMPs have been reported to exert their antimicrobial action through permeabilizing the pathogen membrane and thus their upregulation may aid in the clearing of an infection.

Rapidly increasing antibiotic resistance in already difficult to treat pathogens have prompted a variety of studies employing *G. mellonella* larvae to delineate the efficacy of therapies against these problematic bacteria [66,67,68]. Within these studies, the antibiotic susceptibility profiles of the examined microorganisms are mirroring those established in vitro. Moreover, it has been shown that the MICs of anti-pseudomonal drugs in infected larvae correlated with the susceptibilities in patients [38,52]. We have previously reported the antibacterial capabilities of Mn-tdda-phen, Cu-tdda-phen, and Ag-tdda-phen against *P. aeruginosa* strains originating from CF patients in vitro. While gentamicin was the most effective compound against the reference antibiotic-sensitive strains (ATCC 27853 and PAO1), it had reduced efficacy across the resistant clinical isolates (CF1–CF3). However, the metal-tdda-phen complexes maintained activity that was clinically relevant. Similarities can been seen in the treatment of infected *G. mellonella* larvae. Gentamicin (at the highest tested concentration of 10 µg/larvae) had the greatest potency in treating larvae infected with ATCC 27853 and PAO1 but its activity decreased when administered to larvae infected with the clinical isolates, which had been classified as resistant to the antibiotic [27]. However, and mirroring the in vitro profiles, the activity of gentamicin diminished across the clinical isolates while the activity of the metal-tdda-phen complexes was preserved.

Although definitive treatment with a single agent would be the ideal scenario, due to the expanding resistance profiles of *P. aeruginosa* the empirical administration of antibiotic combinations is utilized by clinicians to control pulmonary exacerbations in CF patients. Across published guidelines, the most common combinations are an aminoglycoside or a fluoroquinolone with an anti-pseudomonal β-lactam, which results in a synergistic bactericidal effect [69]. Gentamicin is a clinically important aminoglycoside antibiotic. It inhibits protein synthesis by binding with a high affinity to the aminoacyl-tRNA site (A site) within the 30S ribosomal subunit, thereby inhibiting the translation process [70]. This produces truncated proteins, affecting the cell wall composition, which increases membrane permeability and subsequently heightens uptake of the drug [71]. However prolonged treatment with gentamicin can have severe adverse effects, such as nephrotoxicity and ototoxicity that are thought to be dose related, with higher concentrations causing greater chance of toxicity. Metal-based drugs have unique mechanisms of action, in comparison to their organic counterparts [14,15,72,73,74,75,76,77]. Such mechanisms include; ligand exchange or release, ROS generation and catalytic generation of toxic species or depletion of essential substrates [72,73]. These mechanisms can be multimodal [75] in nature and are hugely dependent on the metal center and attached ligands [73]. In this study, we assessed the combination of the metal-tdda-phen complexes and gentamicin because they demonstrated synergistic activity against *P. aeruginosa* strains in both planktonic (data not shown) and biofilm forms [27]. The combined therapy of metal-tdda-phen and gentamicin in larvae infected with all strains produced an appreciable increase in survival than those treated with the individual agents, at lower concentrations. Furthermore, combination dampened proliferation of bacteria within the larvae which was elucidated by the larger depletion in bacterial burden in comparison to single treatments. As bacteria adapt to antibiotic treatment, higher doses are required to manage the infection which, as previously mentioned, has been associated with severe adverse effects in patients. The efficacy of the metal-tdda-phen/gentamicin combination at clearing an infection, especially of the resistant clinical isolates, at lower doses is interesting in this regard. Although gentamicin is a bactericidal antibiotic, it is not possible to deduce if the combination is also working in this manner. However, due to the already established antibacterial effects of complex Mn-tdda-phen and its presented capability to induce an immune response through enhancement of hemocytes and immune related genes, it can be postulated that this complex might have several processes for aiding the clearance of an infection.

## 5. Conclusions

In conclusion, the data presented here suggest that Mn-tdda-phen and Ag-tdda-phen are capable of clearing a *P. aeruginosa* infection at concentrations that are non-toxic towards *G. mellonella* larvae. Building on previously published work, the metal-tdda-phen complexes are thought to be multimodal, acting directly on the bacteria but also through stimulating both the cellular and humoral responses that work concomitantly to clear an infection. Although more research is required to understand the mechanisms by which the complexes exert their antibacterial properties, this study highlighted that substituting the metal center alters the toxicity level and immunomodulation properties. Combinations of metal-tdda-phen complexes with gentamicin were able to restore activity of the antibiotic against clinical isolates that were resistant to it, presenting an alternative combination therapeutic approach with greater efficacy.

## Figures and Tables

**Table 1:** Mean larval mortality (%) after 24, 48, and 72 h inoculations with metal-tdda-phen complexes and gentamicin at a concentration range of 2–30 µg/larvae.

Test Complex	Dose µg/Larvae (µM)	Mean Mortality (%) +/− SE over Time (h)		
		24 h	48 h	72 h
Mn-tdda-phen	2 µg (2.71 µM)	0 ± 0	0 ± 0	0 ± 0
	4 µg (5.42 µM)	0 ± 0	0 ± 0	0 ± 0
	10 µg (13.59 µM)	0 ± 0	0 ± 0	0 ± 0
	15 µg (20.39 µM)	0 ± 0	0 ± 0	0 ± 0
	30 µg (40.78 µM)	0 ± 0	6.66 ± 5.77	6.66 ± 5.77
Cu-tdda-phen	2 µg (2.68 µM)	0 ± 0	0 ± 0	0 ± 0
	4 µg (5.36 µM)	0 ± 0	0 ± 0	0 ± 0
	10 µg (13.41 µM)	0 ± 0	0 ± 0	0 ± 0
	15 µg (20.15 µM)	23.33 ± 5.77	46.66 ± 5.77	53.33 ± 5.77
	30 µg (40.3 µM)	76.66 ± 5.77	83.33 ± 5.77	100 ± 0
Ag-tdda-phen	2 µg (1.6 µM)	0 ± 0	0 ± 0	0 ± 0
	4 µg (3.3 µM)	0 ± 0	0 ± 0	0 ± 0
	10 µg (8.3 µM)	0 ± 0	0 ± 0	0 ± 0
	15 µg (12.5 µM)	0 ± 0	0 ± 0	0 ± 0
	30 µg (24.9 µM)	3.33 ± 5.77	23.33 ± 5.77	23.33 ± 5.77
Gentamicin	2 µg (3.5 µM)	0 ± 0	0 ± 0	0 ± 0
	4 µg (6.9 µM)	0 ± 0	0 ± 0	0 ± 0
	10 µg (17.4 µM)	0 ± 0	0 ± 0	0 ± 0
	15 µg (26.1 µM)	0 ± 0	0 ± 0	0 ± 0
	30 µg (52.1 µM)	0 ± 0	13.33 ± 5.77	13.33 ± 5.77
Phen	2 µg (11.1µM)	0 ± 0	0 ± 0	0 ± 0
	4 µg (22.2 µM)	0 ± 0	0 ± 0	0 ± 0
	10 µg (55.5 µM)	53.3 ± 5.8	53.3 ± 5.8	53.3 ± 5.8
	15 µg (83.2 µM)	76.7 ± 5.8	76.7 ± 5.8	76.7 ± 5.8
	30 µg (166.5 µM)	100 ± 0	100 ± 0	100 ± 0
tddaH_2_	2 µg (9 µM)	0 ± 0	0 ± 0	0 ± 0
	4 µg (18 µM)	0 ± 0	0 ± 0	0 ± 0
	10 µg (45 µM)	0 ± 0	0 ± 0	0 ± 0
	15 µg (67.5 µM)	0 ± 0	0 ± 0	0 ± 0
	30 µg (135 µM)	46.7 ± 5.8	46.7 ± 5.8	46.7 ± 5.8

Data are presented as mean ± SE.

**Table 2:** Mean larval mortality (%) after 24, 48, and 72 h inoculation of Mn-tdda-phen and gentamicin, Cu-tdda-phen and gentamicin, and Ag-tdda-phen and gentamicin.

Test Agents	Dose µg/Larvae (µM)	Mean Mortality (%) +/− SE over Time (h)		
		24 h	48 h	72 h
Mn-tdda-phen + Gentamicin	2 µg (2.71 µM) + 2µg (3.5 µM)	0 ± 0	0 ± 0	0 ± 0
	4 µg (5.42 µM) + 4 µg (6.9 µM)	20 ± 5.8	20 ± 5.8	20 ± 5.8
	10 µg (13.59 µM) + 10 µg (17.4 µM)	83.3 ± 3.3	86.7 ± 3.3	86.7 ± 3.3
	2 µg (2.71 µM) + 10 µg (17.4 µM)	43.3 ± 3.3	46.7 ± 3.3	46.7 ± 3.3
	10 µg (13.59 µM) + 2 µg (3.5 µM)	46.7 ± 3.3	50.0 ± 5.8	53.3 ± 3.3
Cu-tdda-phen + Gentamicin	2 µg (2.68 µM) + 2µg (3.5 µM)	26.7 ± 3.3	26.7 ± 3.3	26.7 ± 3.3
	4 µg (5.36 µM) + 4 µg (6.9 µM)	70 ± 5.8	73.3 ± 3.3	73.3 ± 3.3
	10 µg (13.41 µM) + 10 µg (17.4 µM)	100 ± 0	100 ± 0	100 ± 0
	2 µg (2.68 µM) + 10 µg (17.4 µM)	63.3 ± 3.3	66.7 ± 3.3	73.3 ± 3.3
	10 µg (13.41 µM) + 2 µg (3.5 µM)	83.3 ± 3.3	83.3 ± 3.3	83.3 ± 3.3
Ag-tdda-phen + Gentamicin	2 µg (1.6 µM) + 2µg (2.5 µM)	0 ± 0	0 ± 0	0 ± 0
	4 µg (3.3 µM) + 4 µg (6.9 µM)	26.7 ± 3.3	33.3 ± 3.3	33.3 ± 5.8
	10 µg (8.3 µM) + 10 µg (17.4 µM)	83.3 ± 3.3	93.3 ± 6.7	93.3 ± 6.7
	2 µg (1.6 µM) + 10 µg (17.4 µM)	43.3 ± 3.3	53.3 ± 3.3	53.3 ± 3.3
	10 µg (8.3 µM) + 2 µg (2.5 µM)	46.7 ± 6.7	50 ± 5.8	53.3 ± 3.3

Data are presented as mean ± SE.

## Data Availability

The authors confirm that the data supporting the findings of this study are available within the article. However, data can also be found on the TU Dublin central repository, Arrow. If required and the authors can be contacted for further information.

## Supplementary Materials

The following supporting information can be downloaded at: https://www.mdpi.com/article/10.3390/biomedicines10020222/s1, Table S1. Forward and reverse primers for genes related to the immune response of *G. mellonella*.
