# Supplementary material for: In Vivo Activity of Metal Complexes Containing 1,10-Phenanthroline and 3,6,9-Trioxaundecanedioate Ligands against Pseudomonas aeruginosa Infection in Galleria mellonella Larvae
Source: Biomedicines. 2022 Jan 21;10(2):222. doi: 10.3390/biomedicines10020222 (PMC8869450; doi:10.3390/biomedicines10020222)
Supplement: Supplementary file 1 [file biomedicines-10-00222-s001.zip › biomedicines-1521651-supplementary.pdf]

Table S1. Forward and reverse primers for genes related to the immune response of *G. mellonella*.

Gene	Sequence (5′-3′)	Fragment size (base pair)
S7e F<br>S7e R	ATGTGCCAATGCCCAGTTG<br>GTGGCTAGGCTTGGGAAGAAT	131
Transferrin F<br>Transferrin R	CCCGAAGATGAACGATCAC<br>CGAAAGGCCTAGAACGTTTG	535
IMPI F<br>IMPI R	ATTTGTAACGGTGGACACGA<br>CGCAAATTGGTATGCATGG	409
Galiomicin F<br>Galiomicin R	CCTCTGATTGCAATGCTGAGTG<br>GCTGCCAAGTTAGTCAACAGG	359
Gallerimycin F<br>Gallerimycin R	GAAGATCGCTTTCATAGTCGC<br>TACTCCTGCAGTTAGCAATGC	175
